# A cluster randomized controlled trial aimed at implementation of local quality improvement collaboratives to improve prescribing and test ordering performance of general practitioners: Study Protocol

**DOI:** 10.1186/1748-5908-4-6

**Published:** 2009-02-17

**Authors:** Jasper Trietsch, Trudy van der Weijden, Wim Verstappen, Rob Janknegt, Paul Muijrers, Ron Winkens, Ben van Steenkiste, Richard Grol, Job Metsemakers

**Affiliations:** 1 Maastricht University, Dept. of General Practice, School for Public Health and Primary Care (CAPRHI), Maastricht, The Netherlands; 2 GP out-of-hours centre, Den Bosch/Eindhoven, the Netherlands; 3 OWM Centrale Zorgverzekeraars group, Zorgverzekeraar UA, Tilburg, The Netherlands; 4 Maasland Hospital, Sittard, The Netherlands; 5 Diagnostic Centre and department of Integrated Care, Maastricht University Medical Centre, Maastricht, The Netherlands; 6 Radboud University Nijmegen Medical Centre, Centre for Quality of Care Research, Nijmegen, The Netherlands

## Abstract

**Background:**

The use of guidelines in general practice is not optimal. Although evidence-based methods to improve guideline adherence are available, variation in physician adherence to general practice guidelines remains relatively high. The objective for this study is to transfer a quality improvement strategy based on audit, feedback, educational materials, and peer group discussion moderated by local opinion leaders to the field. The research questions are: is the multifaceted strategy implemented on a large scale as planned?; what is the effect on general practitioners' (GPs) test ordering and prescribing behaviour?; and what are the costs of implementing the strategy?

**Methods:**

In order to evaluate the effects, costs and feasibility of this new strategy we plan a multi-centre cluster randomized controlled trial (RCT) with a balanced incomplete block design. Local GP groups in the south of the Netherlands already taking part in pharmacotherapeutic audit meeting groups, will be recruited by regional health officers. Approximately 50 groups of GPs will be randomly allocated to two arms. These GPs will be offered two different balanced sets of clinical topics. Each GP within a group will receive comparative feedback on test ordering and prescribing performance. The feedback will be discussed in the group and working agreements will be created after discussion of the guidelines and barriers to change. The data for the feedback will be collected from existing and newly formed databases, both at baseline and after one year.

**Discussion:**

We are not aware of published studies on successes and failures of attempts to transfer to the stakeholders in the field a multifaceted strategy aimed at GPs' test ordering and prescribing behaviour. This pragmatic study will focus on compatibility with existing infrastructure, while permitting a certain degree of adaptation to local needs and routines.

**Trial registration:**

Nederlands Trial Register ISRCTN40008171

## Background

With the ever-growing volume of evidence from medical research, it has become impossible for physicians to remain fully up to date. Reviews and guidelines therefore summarize large quantities of information, making it more easily available to field workers. In the Netherlands, general practitioners (GPs) now have access to more than 80 evidence-based medical guidelines developed by the Dutch College of General Practitioners (NHG). Although general adherence to these guidelines is approximately 70%, the inter-physician variation is large, and adherence to certain aspects of these guidelines proves to be difficult [1-3]. Although there may be sensible reasons to deviate from guidelines, such as multi-morbidity in a patient, a physician's level of uncertainty tolerance and patients' preferences, there seems to be room for improvement. The inter-physician variation can be regarded as underdiagnosing or undertreating one group of people and at the same time overdiagnosing and overtreating another group, both leading to inappropriate care [4]. There is considerable inter-physician variation in general practice with regard to test ordering and prescribing [5,6].

Many studies have tried to find evidence for effective implementation strategies to improve quality of care. A multifaceted clustered RCT by Verstappen *et al*. aimed at optimizing GPs' test ordering behaviour by means of local quality improvement collaboratives (LQICs), found a decrease of 8 to 12% in test volumes over a period of six months [7]. This strategy was tested using six topics for continuing medical education (CME). Other studies have tested several implementation strategies to improve test ordering and prescribing behaviour. Passive dissemination of guidelines or recommendations does not seem to influence test ordering behaviour. Audit and feedback have often been used and showed mostly a modest effect in terms of influencing test ordering or prescribing. The effect of audit and feedback on adherence to desired practice ranged from -10% to +68% (median +16%) [8-12]. In other studies, the introduction of a problem-based test ordering form proved to be a promising tool to improve test ordering [7,13-18]. Similar effects on volumes of tests ordered as those in the Verstappen study have been found for more or less similar multifaceted implementation strategies [19,20]. Small group peer review using direct individual feedback seemed to reduce inappropriate prescribing [12,21,22]. Lagerlov found a 6 to 13% improvement in adherence to guidelines for the prescription of anti-asthmatic drugs and antibiotics for urinary tract infections in an RCT using reflection on guidelines and prescription feedback in small groups [23].

The Cochrane Effective Practice and Organization of Care group (EPOC) systematically reviews studies on implementation strategies to improve quality of care. Their work has generated the general insight that multifaceted strategies are usually more effective than single interventions [12,24], although this was not entirely confirmed by an NHS HTA review by Grimshaw *et al*. [16]. The prevailing insight is that the effect of an intervention is larger when tailored strategies are used and when barriers to and facilitators of change are addressed.

Grol has identified in his model of effective implementation six stages in quality of care improvement[25]. In the first stage, new research findings, new guidelines, experienced weaknesses, or best practices create an opportunity for quality improvement. In the second stage, after the initial implementation process has been planned, targets for improvement or change are set. Prior to the actual implementation, the performance, target group, and setting are analysed. In the fourth stage, the strategies that are to be used are identified and tested. The implementation is developed, tested, and executed. Finally, the implementation is evaluated and adapted, if necessary [25]. The present study will deal with the actual sustainable transfer of a successful implementation strategy to the field. We are not aware of published studies testing this process, or whether effects are sustainable when transferred to the field. Nor are we aware of published studies on the implementation of a large-scale strategy aimed at influencing both the test ordering and prescribing behaviour of GPs simultaneously, using peer review and social influencing in primary care collaboratives.

In the Netherlands, existing networks of pharmacotherapeutic audit meetings (PTAM) can be used to disseminate and implement guidelines on test ordering and prescribing. The goal of setting up these meetings by primary care providers was to improve the quality of their prescribing behaviour [26]. The local groups usually consist of six to ten GPs with affiliated community pharmacists [27]. During the meetings, they discuss the choice of drugs in the context of a specific illness or disease. In recent decades, this form of CME has gained widespread acceptance amongst GPs and policymakers in the Netherlands. However, these sessions tend to offer little or no room for discussions on test ordering. Because no other system of regular meetings exists, the possible underuse, overuse, and misuse of diagnostic services is not discussed by primary care providers on a regular basis.

The Dutch Institute for the Proper Use of Medicine (DGV) supports and initiates local or regional implementation of quality improvement projects on the use of drugs and supports local PTAM groups by supplying them with information and educational materials [28]. Performance levels of PTAM groups are assessed once a year and rated on the basis of four levels, level one being the poorest level of performance and level four the highest. We will use this division into levels as a parameter for pre-randomization stratification.

Participation in PTAM groups by GPs is facilitated by national and regional support organizations for primary care, as well as by the government and through incentives by insurance companies. Attendance at PTAM meetings is rewarded by accreditation. Currently, approximately 50% of the group meetings reach the desired level of performance described by policymakers [27]. To reach this level, groups must at least use feedback on prescribing, create working agreements, discuss barriers to change, and evaluate working agreements. Most groups are stable and remain together for 10 years or more, with members mostly being replaced gradually [27]. Because of the nature and stability of these groups, they provide an excellent and safe environment for participants to discuss their own behaviour and barriers to change. We expect this existing system of PTAM groups will ensure sustainability of the implementation itself. Therefore, we plan to use these groups in a large pragmatic trial on the implementation of guidelines, using the strategy previously tested by Verstappen *et al*. [7]. However, we will expand the strategy, using social interaction and external influencing as key approaches for establishing behavioural change, to both test ordering and drug prescribing. In our view, the groups will no longer function merely as a PTAM group, but rather begin acting as LQICs. This trial is expected to show whether the effects found in less pragmatic trials can be confirmed. Aiming at both test ordering and drug prescribing, our combination strategy could lead to an even larger effect because of synergy. We will also evaluate the costs of implementing the strategy on a large scale.

## Objectives and research questions

### Hypotheses

We expect that the transfer of the strategy of LQICs to stakeholders in the field will be feasible. We hereby hope to create a solid basis for continuation after the end of the study.

We also expect that large-scale implementation, giving attention to both test ordering and prescribing behaviour, will lead to similar changes in performance as those found on test ordering in the trial by Verstappen *et al*. [7].

Successful implementation will be positively related to the level of group performance of the groups included, in terms of level of attendance, number of meetings, drawing up working agreements, discussing barriers to change, and evaluating working agreements.

### Objectives

1. To implement the LQIC strategy in the south of the Netherlands, stimulating the relevant parties in the field to take the lead.

2. To determine the critical conditions for effective nationwide implementation.

3. To improve the level of group performance in the participating groups.

4. To reduce undesirable physician variation in test ordering and prescribing; and to reduce underuse or overuse of specific tests and drugs.

5. To examine the costs of large-scale implementation of this strategy, and thus to be able to predict future costs for expansion and maintenance of the strategy.

#### Research questions

##### Process

1. Was the strategy implemented as planned?

2. What were the barriers to and facilitators of the implementation of the strategy?

3. Has the level of group performance been improved in the participating groups?

##### Effect

1. Do the volumes of tests ordered and drugs prescribed change in the preferred direction, as described in the working agreements of the LQICs, compared to baseline?

2. What is the effect of this strategy on GPs' test ordering and prescribing behaviour in terms of interphysician variation and total volumes of tests and prescriptions with respect to specific clinical topics, compared to that among GPs exposed to the same strategy but for other topics?

3. Is any gain in the level of group performance predictive of the effect achieved?

##### Cost

What are the costs of implementing the strategy?

## Methods

### Design and ethics

**Figure 1:**
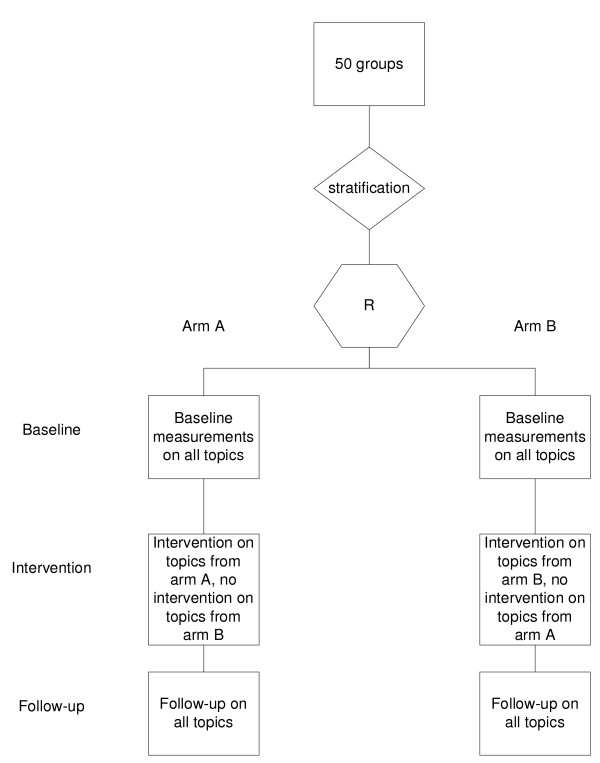
**Flowchart of randomization and intervention**.

This multi-centre study will use a balanced incomplete block design, consisting of two arms (Figure 1). LQICs will be allocated at random to one of these two arms. All LQICs allocated to arm A will receive the intervention with respect to the clinical topics associated with arm A. All LQICs allocated to arm B will receive the same intervention, but with respect to the topics associated with arm B (table 1). Each arm will have five different CME topics to choose from. Each LQIC will choose three different topics for their discussions, and serve as a control for the other arm. The GPs will not be aware of the topics they are serving as controls for, to avoid the Hawthorne effect [29].

**Table 1:** Modules and distribution over the research arms.

	Modules		
	
	Topic	Examples of tests	Examples of drugs
Arm A	Hypercholesterolaemia	LDL	Statines
	
	Anaemia	haemoglobin	ferro medication
	
	Rheumatic complaints	Waaler-Rose	NSAIDs
	
	Urinary tract infections	Urinary cultures	Antibiotics
	
	Prostate complaints	PSA	α-blockers

Arm B	Type 2 Diabetes Mellitus	HbA1c	Metformin
	
	Dyspepsia	gastroscopy	proton-pump inhibitors
	
	Chlamydia infections	chlamydia cultures	Antibiotics
	
	Thyroid problems	TSH	Levothyroxine
	
	Perimenopauzal complaints	FSH	Estradiol

For a complete list of all tests and drugs for the modules [See additional file 2]

The Maastricht Medical Research Ethics Committee has approved this study. All participating GPs will be asked to sign a written informed consent form.

### Population

LQIC groups will be recruited by regional medical coordinators, which are regional health officers or managers often employed by regional hospitals or primary care laboratories. We have identified 24 organizations offering diagnostic facilities in the south of the Netherlands. All organizations will be visited by the researcher and asked to cooperate. Each medical coordinator then will be asked to recruit two to four LQIC groups. They will only be included when all group members consent to participate. The area from which groups can be recruited will be restricted to the three southern provinces of the Netherlands (Limburg, Noord-Brabant, and Zeeland) because these are covered by the insurance companies who provide data for the pharmaceutical database at Maastricht University (UM). A representative with special expertise in and knowledge of diagnostic testing, recruited by the medical coordinator, will attend each LQIC meeting. This representative will receive copies of the feedback forms of all GPs in a LQIC, to enable him or her to prepare the sessions. The representative will act as a moderator during the sessions devoted to diagnostics, after having been trained to do so (see under 'training'). The medical coordinator will finally also liaise between their diagnostic centre and the research team. Other stakeholders in our strategy include community pharmacists, UM, the DGV, insurance companies, PTAM groups, and individual GPs. Community pharmacists play a major role in PTAMs in the Netherlands, providing expertise and sometimes feedback on prescriptions to the participating GPs. Our intervention will leave the role of the pharmacists more or less unchanged. They provide easily accessible knowledge for GPs, thus breaking down barriers which might be inherent in distance support such as academic detailing. Like the medical coordinator, a pharmacist will function as a moderator in the LQIC. All community pharmacists will receive training prior to the first session, as described above. The pharmacists will receive copies of the feedback forms of all participating GPs in a group, to enable them to prepare the sessions.

The initiator of this trial is the Department of General Practice of Maastricht University. The design and maintenance of the database on diagnostics and the data gathering process are coordinated by the first author. The Maastricht University Centre for Information and Data Management (MEMIC) will host the diagnostics database, as they already do for the prescriptions database.

### Randomization

LQIC groups will be randomized as such (cluster randomisation). The intervention is aimed at these groups. Pre-randomization stratification will be performed on group size and level of group performance using a pre-randomization questionnaire [See additional file 1] prior to the intervention. The levels of group performance are as determined by DGV [28]. This level is a known confounder for an effective intervention on medical education among groups of GPs [30,31]. After stratification, all groups within a stratum will be randomly allocated to either arm A or arm B (Figure 1).

### Sample size

A sample size calculation is not really possible beforehand, because it is not yet known what working agreements will be created and with respect to what tests or drugs. The specific targets, incorporated in working agreements will probably be based on extreme overuse or underuse of certain tests or drugs by some or all group members. It is possible, for instance, that the group will decide to eliminate a particular obsolete test or drug or create a working agreement to decrease or increase the mean volume of tests ordered or drugs prescribed by 20%, from 35% to 55%.

The sample size calculation used in this trial is as follows: to detect an improvement of 20% in a certain target between groups, assuming an ICC of 0.10 [5], an alpha of 0.05 and a beta of 0.1 and a mean group size of seven, 44 LQICs would be needed. Anticipating a dropout of 10%, we would need to recruit 50 groups. A population this large would account for approximately 900,000 registered patients.

### Intervention

Several theories have been postulated on how change in healthcare can be accomplished, and how effective change strategies can work in implementation of innovation. In cognitive theories, professional behaviour is considered to result from rational processes and experiences from earlier caseloads. In social interaction theories, change of professional behaviour is thought to be strongly mediated by peers in a group, the strength of inter-individual ties within groups, the existence of opinion leaders, and how much the desired behaviour is consistent with, and fits in, everyday practice. In total quality management theories, the use of systematically gathered data is considered to be crucial to facilitate effective professional development. These data can then be used in plan-do-study-act cycles (PDSA cycles) to provide insight into displayed behaviour and help identify areas where improvement is possible. This leads to the description of targets. These theories may overlap or may be complementary. In implementation science, the use of these theories as a framework is considered obligatory [32]. This intervention therefore will be multifaceted and consist of audit, comparative graphical feedback, and small group work with peer review of each other's performance, discussion of barriers to change, reaching agreement on future policy, and testing the agreement. After randomization to arm A or B, each group can choose from the corresponding set of five clinical topics allocated to that arm, to decide which three topics they want to discuss. Two balanced sets of topics, one for each arm, have been defined by the researchers. Each set consists of three major topics, from which the group has to choose two, and two minor topics, one of which has to be chosen. Thus, each LQIC will be asked to complete the entire strategy for three clinical topics of their choice during the intervention period. They are free to schedule extra meetings on topics not included in this trial, but these meetings will not be included in the final analysis. Feedback on the topic under discussion will be sent to the medical coordinator (diagnostic feedback) or local community pharmacists (prescription feedback) two weeks prior to the test ordering or the prescribing session of the LQIC, together with the relevant educational materials (see under 'clinical topics'). The first session, which will last approximately 90 minutes, will address the diagnostic test ordering behaviour of the individual GPs and will have the structure described under 'session structure' (Table 2). During this session, the GPs will discuss their diagnostic test ordering patterns and relate them to the guidelines provided. Individual and group working agreements will be created after barriers to change have been discussed. The second session will have the same structure, but the subject for discussion will be physicians' prescribing performance. This session will end by creating group and individual working agreements about preferred medication. Barriers to change from an individual perspective will again have to be discussed. After this first topic has been completed, the cycle will be repeated, for a new topic, as shown in Table 3. At the start of this new cycle, the group will reflect on the previous agreements, and revise them if necessary. The working agreements will then be prepared for further dissemination in the practices. Each session will be chaired by a member of the LQIC itself. When test ordering is discussed, a local representative from the diagnostic centre will be present, while a local community pharmacist will be present when pharmacotherapy is discussed. They will act as moderators, not as chairpersons.

**Table 2:** Session structure

90 minutes	5 min	Explaining the method/reflection on previous topic
	
	5 min	Critical look at participants' own feedback
	
	5 min	Pairwise/group discussion on inter-individual differences
	
	25 min	Plenary discussion, relating feedback to guidelines
	
	10 min	Pairwise discussion on barriers to change
	
	25 min	Plenary discussion on barriers to change, aimed at problem solving
	
	15 min	Drawing up individual and group working agreements

**Table 3:** Example of a schedule for the intervention

Topic	GPs	Medical coordinator	Community pharmacist
1. Anaemia	1. Meeting on tests	Moderator	Prepares second session
	
	2. Meeting on drugs	Prepares third session	moderator

2. Chlamydia infections	3. session on test and drugs (anaemia) 1. session on tests (Chlamydia)	moderator	Present as expert

We will test the model and the logistics needed prior to the large-scale implementation. We plan to do this in a small pilot study involving five groups of GPs. This pilot study will run for four months, during which period the participating GP groups will schedule two meetings. Each session will be structured according to the method provided by the researchers. The first session will address test ordering, while the second session will address prescribing. For reasons of efficiency, a set of only three topics will be used for the pilot study. The topics, which have been proposed by the project team members, are anaemia, dyspepsia, and asthma in combination with chronic obstructive pulmonary disease (COPD).

### Clinical topics

The set of clinical topics the GP groups can choose from in the main study has been proposed by the authors. After eligible topics were selected and divided over the two trial arms, both arms were balanced in terms of the weight of the topics. The weight depends on the prevalence of the underlying disease and whether the emphasis within the topic is on either the volume of tests ordered or the drugs prescribed. The two sets of topics are also balanced in terms of subjects, emphasising diagnostic or prescribing features (Table 1). Each topic includes a number of tests [See Additional file 2] and drugs [See Additional file 3] predefined by the project group. For the purpose of feedback and education, these include both well-accepted and commonly not accepted (or even obsolete) tests and drugs. Educational materials on each topic will be based on the relevant national primary care guidelines from the Dutch College of General Practitioners, guidelines from the Dutch Institute for Healthcare Improvement (CBO), and international guidelines if applicable. Guidelines will be read and 'condensed' into short versions called modules. These modules have been drafted by one of the authors (JT) and then commented on by an expert on the topic. Indicative prices for each test and drug will be provided, as well as a short description of its values and drawbacks, given the indication. Each module will consist of a maximum of six easily searchable pages.

### Extraction of feedback data

**Figure 2:**
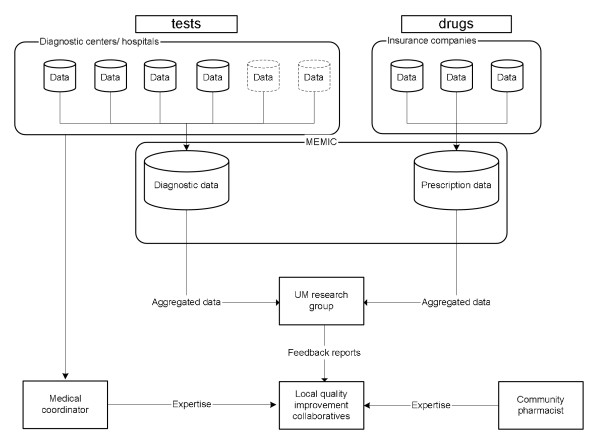
**Data and Knowledge flowchart**.

**Figure 3:**
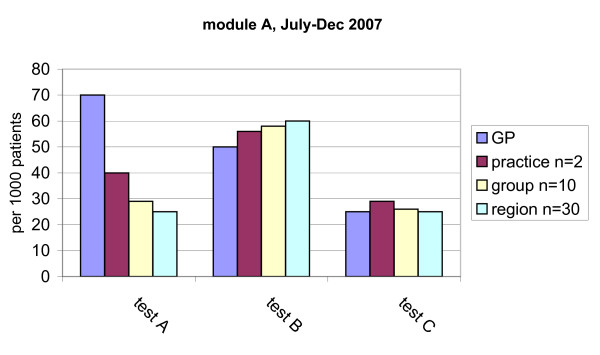
**Example of a graphical comparative feedback sheet**.

Data on test ordering behaviour will be extracted by the regional coordinators from the various databases available at the participating hospital laboratories or primary care diagnostic centres. Each centre will receive a data fact sheet prescribing the required data format. This format is based on rational criteria for laboratory test registration to facilitate the integration of the individual databases into one main database. All datasets on diagnostics will be combined into one newly formed database, to be maintained by UM (Figure 2). Data on prescribing behaviour will be extracted from the databases of health insurers and collected into one database, as has already been done at our institute. This database consists of the reimbursements for prescriptions written by GPs for approximately 5.5 million persons in the south of the Netherlands. Feedback will then be derived from the two main databases and processed into graphical comparative feedback reports. Data will be presented as the volume of tests ordered (*e.g*., haemoglobin) or defined daily dosages (DDDs) prescribed per 1000 patients per six months. Participating GPs will receive their data as clustered column charts, each cluster presenting the data for the individual GP, the practice in which he or she works, the small group and the wider region. An example of such a graphical feedback report is shown in Figure 3.

### LQIC meeting structure

Each meeting will be structured according to a uniform schedule. After participants have studied the feedback in pairs or as a group, they will discuss it. Subsequently, the guidelines as described in the educational materials will be discussed in relation to the feedback. A plan will then be formulated to improve the test ordering or prescribing behaviour. The next step will involve addressing and discussing all the barriers to change at individual and group levels. Finally, working agreements will be created regarding test ordering and prescribing behaviour for the tests and drugs discussed. A standardized group meeting structure card will be provided to each LQIC, showing the structure as recommended by the researchers. However, groups will be free to adapt the structure to their own preferences or needs.

### Training

The participating medical coordinators and local community pharmacists will be trained prior to the first LQIC session, in a two- to three-hour standardized training session covering three main subjects. The first subject will involve an explanation of the structure of the trial, the objectives, the development of the outlines, the source of the feedback data, and the process of data gathering. The second subject will be the preferred structure for the meetings, the tools that are to be used, how to read the feedback reports and relate the feedback to the guidelines. The final subject of the training session will be how to act as a moderator instead of a chair during a meeting. Training sessions will partially be constructed like a LQIC meeting, with the trainees acting as GPs and the trainer as the moderator.

### Variables

#### Outcome measures

#### Process evaluation

1. The performance level of the small group collaborative.

2. Process data such as attendance at meetings, actually creating working agreements, following the LQIC strategy, the number of groups that complete participation, and the number of regions actually participating.

#### Effect evaluation

1. The volumes of particular tests ordered and particular drugs prescribed for which the group has agreed that change, either decrease or increase, would be necessary.

2. The total volumes of tests ordered and drugs prescribed by the participating GPs for the clinical topics chosen.

3. The inter-physician variation in test ordering and prescribing behaviour for the clinical topics chosen.

#### Cost evaluation

The costs of implementing the LQIC strategy.

### Explanatory variables

We will monitor data that are known to moderate quality assurance strategies. Therefore the following data will be gathered prior to the intervention: group size, age and gender of GPs, type of practice, number of patients registered with the practice, number of patients a GP is accountable for, number of working hours a week per GP, number of working hours a week for the group practice as a whole, distance to the hospital/diagnostic centre, responsibility for training GP trainees, total number of GPs collaborating in the practice, whether a GP admits sales representatives from pharmaceutical firms and if so how often, involvement in developing national guidelines, and GPs field(s) of special expertise.

All medical coordinators will be asked if problem-based test ordering forms are used in their region and to send us a copy of such a form.

### Measurements

Prior to randomization, the chair of the group will be asked to fill out a short pre-randomization form, with which we will be able to determine the number of GPs in the group and be able to assess the level of group performance [See Additional file 1]. Data on test ordering and prescribing behavior will be extracted from the existing databases at baseline (t = 0) and t = 6 months, t = 12 months and t = 18 months. The dataset obtained at t = 0 and the final set will be used for a before-after analysis. A new questionnaire will be sent to the chair, assessing the level of group performance after the intervention. After each meeting, the chair will be asked to fill out a form with questions about the structure of the session, whether working agreements were created, whether barriers to change were discussed and if so what the nature of these barriers was, what educational materials were used, and the group members' experiences with the strategy.

The process of implementing the strategy in the south of the Netherlands will be monitored. Participants will be questioned about their experiences with the strategy. Participating GPs will be asked to report their experiences with the strategy, and to provide us with the necessary details on the sessions they have attended. After each session, the targets set by each group will be recorded.

### Analysis

Analysis will be based on the intention-to-treat principle. Data on GPs lost to follow-up will be extracted from the various databases if possible.

We will analyse covariance using test and drug volumes during the intervention period as the dependent variables, and the baseline data and the explanatory variables as independent variables. The analysis will be repeated using proportions stemming from prescription performance indicators, if available. The unit of allocation to the trial is the LQIC. In larger practices with more than one GP, not all volumes of tests ordered and drugs prescribed will be traceable to an individual GP. In these cases, the unit of analysis will be the practice as a whole. Because of this unit of analysis error, the data will be analysed using multilevel modelling. Data on drugs and tests will be clustered to individual GPs at level one, the practice at level two, the LQIC at level three and the region at level four.

The nature of this study makes it difficult to blind the participants, except for the tests or drugs serving as controls in the other arm. The data analyst will be blinded for the allocation result. Costs of the intervention will be calculated. A cost-effectiveness analysis will be based on these figures. We will use cost minimization analysis from a societal perspective, assuming that the strategy will reduce redundant testing and prescribing. If there are signs of improvement of care (higher scores on the performance indicators), the impact on health may be estimated by modelling the future gains and benefits. Data include costs of coordinating the strategy by the regional contact group, of preparing feedback reports and of chairing the GP groups. The costs of the entire test ordering strategy by Verstappen were €554.70 per GP per six months (three meetings). The major part of the cost of this strategy consisted of opportunity costs, *viz *the costs of the GPs' time spent attending the session. Because GPs were already attending these meetings and were financially compensated, it seems fair to ignore the opportunity costs. This results in costs of the test ordering strategy of €92.70. The gains obtained by improving test ordering behaviour were €301.00 per GP per six months. Introducing the test ordering strategy would save €208.30 (92.70 to 301.00) per GP per six months [33]. Because prescribing costs are higher, the cost reductions gained by reducing superfluous prescribing should also be higher.

### Time schedule

The intervention period will start in September 2007 and run through the spring of 2009. Process evaluation will start when all groups are included. During the intervention, new datasets will be obtained every six months in order to keep the databases up-to-date for future use in new sessions.

## Discussion

To our knowledge, few studies have been published on the transfer of effective implementation strategies to the field. Our strategy has proved to be effective in an earlier trial on test ordering by GPs in the Netherlands. However, because this strategy was disseminated and controlled by academics, it remains unclear how large its effect will be when transferred to the field. We set up a pragmatic design in order to test this final step in implementation research, giving the diagnostic centres a leading role and leaving GPs much room to adapt and to internalise the strategy. The project team will act as facilitators to these centres, the pharmacists involved, and the LQICs. The strategy is targeted first on test ordering and second on prescribing, which is the natural order followed by GPs when consulted by a patient.

Our strategy is based upon several theories on effective behaviour change and on effective implementation. These theories can be identified at several levels of organisation in our trial. At the level of diagnostic centres and the LQICs, we expect the innovators and early adopters to join the trial, which refers to Roger's innovation-diffusion theory [34]. Within groups we expect to see change according to theories such as Ajzen's theory of planned behaviour and the PDSA-cycles [25,35]. During a meeting, we expect to see the preparation for change based on performance data and actual actions towards change. When new data will be provided to the groups, we expect reflection on the goals previously set. The theory of planned behaviour states that individuals are willing to show change in behaviour dependent on the perceived control over the behaviour itself, the attitude of the individual to the desired behaviour, and the perceived social norms. By providing graphical comparative feedback, we target at these perceived social norms. Comparative feedback sets the norm for a group, and through the phenomenon that one does not like to be an outlier we expect regression to the mean with regard to the inter-physician variation. The moderator who is also an expert on the subject under discussion is expected to act as opinion leader. Furthermore, even a GP from within the group itself can act as a local opinion leader and thus influence the rest of the group.

The existing PTAM group structure in the Netherland is widespread and functions reasonably well. However the need to improve the functioning of these groups is clearly present. Our strategy is known to improve test ordering behaviour of GPs, but is not used widely. Transferring PTAM groups into LQICs gives us the opportunity to add a discussion on test ordering behaviour to existing discussions on prescribing by GPs in PTAMs. The constitution of LQICs therefore is not 'old wine in new bottles' but a completely new approach within existing structures.

Several methodological challenges were encountered when we designed this trial. First, individual GPs are known to choose topics for CME in which they already show good performance [36]. This might result in a 'ceiling effect', meaning that little or no improvement in test ordering or prescribing behaviour would be possible. However, because the LQIC will have to reach consensus on the clinical topics they choose, the risk of such a ceiling effect is probably not very great.

Second, using an implementation strategy on ten different clinical topics from which GPs can choose introduces challenges to the sample size calculation. We chose to leave the LQICs some freedom of choice with regard to the topics. All clinical topics are well-described in the national guidelines for each topic. We will use a set of 204 tests and drugs to generate feedback [See Additional files 2] [See additional file 3]. Because we do not know what agreements local groups will come to, and do not know beforehand what the desired direction for change is, sample size calculation is very difficult. Because we intend to improve care by using the national guidelines, we do not expect to decrease quality of care by this study. However, it is impossible to predict if change will be towards better care.

Third, the databases we use are complex, as are the origins of the data. Most local databases on diagnostics used in this trial are intended primarily for billing purposes. This might create problems when extracting data, reading it into a central database and translating it into feedback. In the past, no significant problems were encountered when extracting data from laboratories (personal communication by Verstappen). Data on tests not performed within a laboratory (*e.g*., gastroscopy and X-rays), however, are often stored in separate databases and might not be linked to a GP but to a patient. In these cases, tracing the GP who ordered the test is possible but will require an extra effort from the diagnostic centres. It is possible that recruiting groups, supplying a moderator for the sessions and implementing this time-consuming data extraction process might prove to be too much of an effort for the centres. Most contact persons of the centres, however, have indicated that they were most willing to cooperate and were aware of the opportunities offered by this trial.

Fourth, the database on prescriptions consists of data from the large insurance companies in the south of the Netherlands. Using these records as a basis for feedback might create several problems. Although most inhabitants of the southern provinces are insured by one of these companies, prescription data for patients insured with other companies will not be included in our database on prescriptions. This problem might be solved in the future by adding more insurance companies to the database. Another potential problem may be that recording errors are likely to be present in the databases. Desk staff at local pharmacies often links a prescription to one of the GPs in a practice, and often almost all prescriptions for a practice are thus linked to one physician, even when several physicians collaborate in the practice. This creates an inaccuracy in the database, but only for GPs sharing an office. To solve this problem, we will also aggregate to an extra level in these cases, *viz *the subgroup of GPs sharing an office, thus creating a fourth column on the graphical feedback sheet. The last problem we expect to encounter using a large database on prescription is that we do not know the indication for which medication was prescribed; these indications are not known to pharmacists and thus are not stored in any database. This makes it impossible to trace a prescription back to a specific disease. By building a similar database on tests ordered by GPs, we will encounter this problem as well. We do not however expect this to be a problem because we will use graphical comparative feedback. All data from all participating GPs are expected to be equally be affected by this problem and thus the feedback will be comparable.

Fifth, the tests of the diabetes and hypercholesterolemia topics partly overlap. We accepted this, however, because in diabetes, the glucose and HbA1c items are the primary indicators, whereas cholesterol, LDL, HDL, and the ratio are the primary indicators in the hypercholesterolemia topic.

Sixth, we have to be aware of the Hawthorne effect. As discussed above, we chose to use a balanced incomplete block design to overcome this problem. The complexity of the strategy, however, would make it more attractive to use a different design and start the trial in phases. This would mean that different regions would enrol in the strategy successively, so we could learn from the early regions what the weaknesses of our design were and what we would have to alter. This would create an opportunity to ameliorate the strategy with each new phase. To this end, a dynamic wait-listed design could have been more appropriate and beneficial [37]. Conversely, we would then have had to wait after completing enrolment and intervening in one region for new data to be added to the database. The delay would be six months after each region. This left us with no choice but to start with the entire population in the same period. In this situation, we considered the balanced incomplete block design to be most useful.

Finally, GPs and moderators cannot be sufficiently blinded in our present design. However, because GPs do not know what clinical topics are available in the arm they are not allocated to, we do achieve some level of blinding.

Notwithstanding these methodological challenges, there are also opportunities in the Dutch healthcare system that make it attractive to start this trial now. First, the strategy we intend to use fits in well with the new Dutch healthcare system. After the recent reform, healthcare has turned into a competitive business, in which financial profits and market shares may influence decision-making. Our study might create profiling opportunities for centres, which might bind GPs more tightly to them, and thus might be a way for the centres to improve their chances in this market. Finally, diagnostic centres are under increasing pressure from various parties in the healthcare system to provide feedback to GPs. GPs want feedback to monitor and claim results when treating chronically ill patients (*e.g*., diabetics), while insurance companies want laboratories to provide feedback in order to influence test ordering behaviour, and primary care organizations need GPs' performance data for various reasons, such as certification.

A preliminary investigation identified 24 eligible diagnostic centres in hospitals, all of which provide diagnostic facilities to GPs. All were contacted and appointments for personal visits were made. Two centres were not interested in participating, and were therefore not visited. Two centres expressed an interest but faced major strategic challenges and found no time to participate. The remaining 20 centres all agreed to participate. One of the participating centres will not be asked to recruit groups, however, because it is not linked to a region like the other centres, which means that knowledge of local PTAM group structures is lacking. This centre will, however, participate in the large database on diagnostics.

In the south of the Netherlands, health insurance is offered predominantly by two companies, which insure the majority of the inhabitants of these provinces. These insurance companies regularly send updated reports on prescription data to UM. These files are and will be combined into one research database on prescriptions, maintained by MEMIC. Because the recent health care reform in the Netherlands, insurance companies have been given a large role in guarding and improving the quality and continuity of care. They promote the existence of PTAM groups in order to improve the quality of care, giving financial incentives to GPs for attending such group meetings. In some cases, extra incentives are given if working agreements are created and adhered to. However, the insurers are unable to evaluate the quality of the group work. The strategy evaluated in the proposed study should provide them with a tool to ensure high quality group meetings.

## Competing interests

The authors declare that they have no competing interests.

## Authors' contributions

JT drafted the manuscript, participated in its design and is the researcher on the trial. TvdW conceived of the study, participated in the design and coordination and helped to draft the manuscript. WV, RJ, PM, JM, BvS and RG all conceived of the study, participated in its design and read and corrected earlier versions of the manuscript. RW read and corrected earlier versions of the manuscript. All authors have read and approved the final manuscript. The Additional Files below (4, 5 and 6) refer to information regarding  The Funding approval ZonMw, The Approval ethical committee and The CONSORT Cluster RCT Checklist. 

## Supplementary Material

Additional file 1 ** This file displays the pre-randomization questionnaire as it was sent to the chair of each LQIC.** Click here for file

Additional file 2 **The impact of local quality improvement collaboratives additional file 2.** This file includes all the diagnostic tests used in this trial, the diversion over the modules and how each item is labelled on the feedback form. Click here for file

Additional file 3 **The impact of local quality improvement collaboratives additional file 3.** This file includes all the farmaceuticals used in this trial, the diversion over the modules and how each item is labelled on the feedback form. Click here for file

Additional file 4 **Funding approval ZonMw.** Scanned letter of ZonMw in which the funding of this trial is confirmed. Click here for file

Additional file 5 **Approval ethical committee.** Scanned letter of the Maastricht ethical committee, stating that it is not required to fully review this trial by the committee because Dutch law on research with humans is not applicable. Click here for file

Additional file 6 **CONSORT Cluster RCT Checklist. checklist with reference to page numbers in this trial concerning the CONSORT statement for cluster-RCTs.** Click here for file
