# Supplementary material for: A cluster randomized controlled trial aimed at implementation of local quality improvement collaboratives to improve prescribing and test ordering performance of general practitioners: Study Protocol
Source: Implement Sci. 2009 Feb 17;4:6. doi: 10.1186/1748-5908-4-6 (PMC2656449; doi:10.1186/1748-5908-4-6)
Supplement: Additional file 1 — This file displays the pre-randomization questionnaire as it was sent to the chair of each LQIC. [file 1748-5908-4-6-S1.pdf]

All questions below concern the period from January till December 2007

1. **How many meetings were scheduled for your PTAM group in 2007?**

   ……… meetings (when 0 meetings were scheduled, this was the last question)

2. **On average, how much time was spent on each meeting?**

   ……….hours ……….minutes

3. **Who usually prepared the meetings in 2007?**
   Choose the answer that is most appropriate
   - ☐ general practitioner and pharmacist together
   - ☐ general practitioner or pharmacist
   - ☐ general practitioner
   - ☐ pharmacist
   - ☐ hospital deployed medical specialist
   - ☐ no one
   - ☐ others, i.e. …………………………

4. **Did these people discuss the preparation of the PTAM during a pre-PTAM meeting discussion?**
   - ☐ no, never
   - ☐ yes, but for less than 50% of all PTAM meetings
   - ☐ yes, for more than 50% of all PTAM meetings, but not for all
   - ☐ yes, always

5. **Did you use prescribing feedback or benchmark data during PTAM meetings?**
   - ☐ No, never
   - ☐ yes, but in less than 50% of all PTAM meetings
   - ☐ yes, in more than 50% of all PTAM meetings, but not in all
   - ☐ yes, always

6. **Did the group reach agreement on preferred drugs during the PTAM meetings?**
   - ☐ No, never (if so, this was the last question)
   - ☐ Yes, on one clinical topic
   - ☐ Yes, on two or more clinical topics
   - ☐ Yes, always

7. **Were agreements on the prescribing of drugs recorded in a meeting report?**
   - ☐ No, never
   - ☐ Yes, some but not all agreements
   - ☐ yes, all agreements were recorded in a report

8. **Were targets set by the group?**
   - ☐ No, never
   - ☐ yes, but in less than 50% of all PTAM meetings
   - ☐ yes, in more than 50% of all PTAM meetings, but not in all
   - ☐ yes, always

9. **Were the effects of the agreements on prescribing behaviour evaluated in another PTAM meeting?**
   - ☐ yes
   - ☐ no (if no, this was your last question)

10. Were prescribing feedback or benchmark data used in this meeting?

- ☐ yes
- ☐ no
