# Supplementary material for: A cluster randomized controlled trial aimed at implementation of local quality improvement collaboratives to improve prescribing and test ordering performance of general practitioners: Study Protocol
Source: Implement Sci. 2009 Feb 17;4:6. doi: 10.1186/1748-5908-4-6 (PMC2656449; doi:10.1186/1748-5908-4-6)
Supplement: Additional file 2 — The impact of local quality improvement collaboratives additional file 2. This file includes all the diagnostic tests used in this trial, the diversion over the modules and how each item is labelled on the feedback form. [file 1748-5908-4-6-S2.pdf]

module	test	label on feedback form
diabetes type 2	glucose random	glucose f/ nf
	glucose non-fasting	glucose f/ nf
	glucose fasting	glucose f/ nf
	HbA1c	HbA1c
	total cholesterol	T-cholesterol
	HDL	HDL
	LDL	LDL
	triglycerides	triglyc
	creatinine	creat
	albumin concentration, urine	albumin urine
dyspepsia	^{13}C-ureum breath test	breath test
	^{14}C-ureum breath test	breath test
	faeces test HP	faeces test HP
	faeces test HP (PCR)	feces test HP
	helicobacter pylori, IgG	antibodies HP
	helicobacter pylori, any antibodies	antibodies HP
	helicobacter pylori, IgM	antibodies HP
	gastroscopy	gastroscopy
	X-ray, stomach	X-ray, stomach
anaemia	Hb	Hb/ Ht
	Ht	Hb/ Ht
	anaemia diagnostic cascade	anaemia cascade
	ferritin	ferritin
	bilirubin	bilirubin
	folic acid	folic acid
	vitamin B12	vit B12
	reticulocytes	reticulocytes
	serum iron	serum iron
	LDH	LDH
	total iron binding capacity	transferrin/TIBC
	transferrin	transferrin/TIBC
chlamydia	chlamydia DNA test, urine (PCR)	chlamydia urine
	chlamydia DNA test, endocervical (PCR)	chlamydia endocervical/vaginal
	chlamydia DNA test, vaginal swab (PCR)	chlamydia endocervical/vaginal
	antibodies chlamydia trachomatis, IgA	antibodies CT
	antibodies chlamydia trachomatis, IgG	antibodies CT
	antibodies chlamydia trachomatis, any	antibodies CT
	antibodies chlamydia trachomatis, IgM	antibodies CT
prostate complaints	creatinine	creat
	PSA	PSA
	PSA complex	PSA
	free/total PSA ratio	F/T-PSA
rheumatic complaints	Waaler-Rose	(semi-)qualitative rheumatic disease markers
	Latex test	(semi-)qualitative rheumatic disease markers

	IgM rheumatic disease markers	IgM rheumatic disease markers
	anti-cyclic citrulline peptide antibodies	anti-CCP
	ESR	ESR
	CRP	CRP
	X-ray wrist	X-hand/foot/wrist
	X-ray MTP	X-hand/foot/wrist
	antistreptolysin antibodies titer	AST
thyroid disease	TSH	TSH
	thyroid diagnostics (cascade)	thyroid cascade
	FT4	FT4
	T3	T3
	TSH-receptor stimulating antibodies	TSI
	thyroid peroxidase (TPO) antibodies	TPO-Ab
	ESR	ESR
	leucocytes	luco diff
	leucocyte differentiation	luco diff
	thyroid ultrasound	US thyroid
UTI	urine culture and susceptibility testing	Urine culture
dyslipidaemia	total cholesterol	T-cholesterol
	HDL	HDL
	LDL	LDL
	triglycerides	triglycerides
	total cholesterol/HDL ratio	ratio T-chol/HDL
	homocystein	homocystein
perimenopausal complaints	follicle-stimulating hormone (FSH)	FSH
	luteinizing hormone (LH)	LH
	estradiol	estradiol
