# Supplementary material for: A cluster randomized controlled trial aimed at implementation of local quality improvement collaboratives to improve prescribing and test ordering performance of general practitioners: Study Protocol
Source: Implement Sci. 2009 Feb 17;4:6. doi: 10.1186/1748-5908-4-6 (PMC2656449; doi:10.1186/1748-5908-4-6)
Supplement: Additional file 3 — The impact of local quality improvement collaboratives additional file 3. This file includes all the farmaceuticals used in this trial, the diversion over the modules and how each item is labelled on the feedback form. [file 1748-5908-4-6-S3.pdf]

Module	Drug	ATC-code	Label at feedback form
diabetes type 2	metformin	A10BA02	metformin
	glicazide	A10BB09	glicazide
	glimepiride	A10BB12	glimepiride
	tolbutamide	A10BB03	tolbutamide
	glibenclamide	A10BB01	glibenclamide
	rosiglitazone	A10BG02	rosiglitazone
	pioglitazone	A10BG03	pioglitazone
	repaglinide	A10BX02	other oral blood glucose lowering drugs
	acarbose	A10BF01	other oral blood glucose lowering drugs
	glimepiride/rosiglitazone	A10BD04	combinations, oral
	metformin/glibenclamide	A10BD02	combinations, oral
	metformin/rosiglitazone	A10BD03	combinations, oral
	insulin, fast-acting	A10AB	insulin, fast-acting
	insulin, intermediate-acting	A10AC	Insulin, intermediate-acting
	insulin, intermediate-acting combined with long-acting	A10AD	insulin mix
	insulin, long-acting	A10AE	insulin, long-acting
dyspepsia	magnesium compounds	A02AA	antacids
	auminium compounds	A02AB	antacids
	combinations and complexes of al- ca- en mg-compounds	A02AD	antacids
	antacids with sodium bicarbonates	A02AH	antacids
	famotidine	A02BA03	other H2-antagonists
	ranitidine	A02BA02	ranitidine
	cimetidine	A02BA01	other H2-antagonists
	nizatidine	A02BA04	other H2-antagonists
	omeprazole	A02BC01	omeprazole
	pantoprazole	A02BC02	pantoprazole
	lansoprazole	A02BC03	lansoprazole
	rabeprazole	A02BC04	rabeprazole
	esomeprazole	A02BC05	esomesoprazole
	misoprostol	A02BB01	misoprostol
	Pantopac ®	A02BD04	Pantopac ®
anaemia	ferrous sulfate	B03AA07	ferrous sulfate
	ferrous fumarate	B03AA02	ferrous fumarate
	ferrous gluconate	B03AA03	ferrous gluconate
	ferrous chloride	B03AA05	ferrous chloride
	cyanocobalamin	B03BA01	vitamin B12
	hydroxocobalamine	B03BA03	vitamin B12
	folic acid	B03BB01	folic acid
chlamydia	Doxycycline	J01AA02	doxycycline
	azithromycin	J01FA10	azithromycin
	amoxicillin	J01CA04	amoxicillin
	erythromycin	J01FA01	erythromycin
prostate complaints	alfuzosin	G04CA01	alfuzosin
	tamsulosin	G04CA02	tamsulosin
	doxazosin	C02CA04	other alpha-adrenoreceptor antagonists
	terazosin	G04CA03	other alpha-adrenoreceptor antagonists
	prazosin	C02CA01	other alpha-adrenoreceptor

			antagonists
	finasteride	G04CB01	finasteride
	dutasteride	G04CB02	dutasteride
rheumatic complaints	phenylbutazone	M01AA01	other NSAIDs
	indometacin	M01AB01	indomethacine
	diclofenac	M01AB05	diclofenac
	sulindac	M01AB02	other NSAIDs
	aceclofenac	M01AB16	other NSAIDs
	meloxicam	M01AC06	preferential cox-2 inhibitors
	piroxicam	M01AC01	other NSAIDs
	tenoxicam	M01AC02	other NSAIDs
	ibuprofen	M01AE01	ibuprofen
	naproxen	M01AE02	naproxen
	ketoprofen	M01AE03	other NSAIDs
	flurbiprofen	M01AE09	other NSAIDs
	tiaprofenic acid	M01AE11	other NSAIDs
	dexibuprofen	M01AE14	other NSAIDs
	dexketprofen	M01AE17	other NSAIDs
	tolfenamic acid	M01AG02	other NSAIDs
	azapropazone	M01AX04	other NSAIDs
	diflusinal	N02BA11	other NSAIDs
	metamizole sodium	N02BB02	other NSAIDs
	celecoxib	M01AH01	coxibs
	etoricoxib	M01AH05	coxibs
	nabumetone	M01AX01	preferential cox-2 inhibitors
	diclofenac + misoprostol	M01AB55	diclofenac+ misoprostol
	sulfasalazine (text no feedback)	A07EC01	DMARDs
	methotrexate (text no feedback)	L01BA01	DMARDs
	leflunomide (text no feedback)	L04AA13	DMARDs
	etanercept (text no feedback)	L04AA11	DMARDs
	infliximab (text no feedback)	L04AA12	DMARDs
	gold preparations (text no feedback)	M01CB	DMARDs
	penicillamine (text no feedback)	M01CC01	DMARDs
	chloroquine (text no feedback)	P01BA01	DMARDs
	azathioprine (text no feedback)	L04AX01	DMARDs
	ciclosporin (text no feedback)	L04AD01	DMARDs
thyroid disease	levothyroxine sodium	H03AA01	levothyroxine
	liothyronine sodium	H03AA02	liothyronine
	thiamazole	H03BB02	thiamazole
UTI	nitrofurantoin	J01XE01	nitrofurantoin
	trimethoprim	J01EA01	trimethoprim
	fosfomycin	J01XX01	fosfomycin
	amoxicillin and enzyme inhibitor	J01CR02	amoxicillin/enzyme inhibitor
	sulfamethoxazole and trimethoprim	J01EE01	co-trimoxazole
	norfloxacin	J01MA06	norfloxacin
	ciprofloxacin	J01MA02	ciprofloxacin
dyslipidaemia	simvastatin	C10AA01	simvastatin
	pravastatin	C10AA03	pravastatin
	atorvastatin	C10AA05	atorvastatin
	fluvastatin	C10AA04	fluvastatin
	rosuvastatin	C10AA07	rosuvastatin
	bezafibrate	C10AB02	fibrates
	gemfibrozil	C10AB04	fibrates

	ciprofibrate	C10AB08	fibrates
	acipimox	C10AD06	nicotinic acid (-analogue)
	ezetimibe	C10AX09	ezetimibe
	xantinol nicotinate	C04AD02	nicotinic acid (analogue)
	nicotinic acid	C10AD02	nicotinic acid (analogue)
	colestyramine	C10AC01	colestyramine
	ezetimibe / simvastatin	C10AX	combinations
perimenopausal complaints	estradiol	G03CA03	estrogen monotherapy
	estriol	G03CA04	estrogen monotherapy
	ethinylestradiol	G03CA01	estrogen monotherapy
	conjugated estrogens	G03CA57	estrogen monotherapy
	prostagens and estrogens, fixed comb.	G03AA	oral contraceptives, >50 yr
	prostagens and estrogens, fixed comb.	G03AB	oral contraceptives, >50 yr
	prostagens and estrogens, fixed comb.	G03AC	oral contraceptives, >50 yr
	prostagens and estrogens, fixed comb.	G03BB01	oral contraceptives, >50 yr
	estrogen/cyproterone (Climene '28' ®)	G03HB01	combinations with estrogens
	estrogen/drospirenone (Angeliq ®)	G03FA17	combinations with estrogens
	estrogen/dydrogesterone, sequential (Femoston®)	G03FB08	combinations with estrogens
	estrogen/dydrogesterone, fixed (Femoston continu ®)	G03FA14	combinations with estrogens
	estrogen/norethisterone, sequential (Trisequens ®)	G03FB05	combinations with estrogens
	estrogen /norethisterone, fixed (Activelle ®, Kliogest ®)	G03FA01	combinations with estrogens
	estrogen /norethisterone, patch (Estracomb TTS ®)	G03FB05	combinations with estrogens
	clonidine	C02AC01	clonidine
	tibolone	G03CX01	tibolone
