# Supplementary material for: A cluster randomized controlled trial aimed at implementation of local quality improvement collaboratives to improve prescribing and test ordering performance of general practitioners: Study Protocol
Source: Implement Sci. 2009 Feb 17;4:6. doi: 10.1186/1748-5908-4-6 (PMC2656449; doi:10.1186/1748-5908-4-6)
Supplement: Additional file 4 — Funding approval ZonMw. Scanned letter of ZonMw in which the funding of this trial is confirmed. [file 1748-5908-4-6-S4.pdf]

ZonMw

Universiteit Maastricht
Faculteit der Gezondheidswetenschappen
Gezondheidsethiek en Wijsbegeerte
Prof. dr. G.A.M. Widdershoven
Postbus 616
6200 MD MAASTRICHT

Laan van Nieuw Oost Indië 334
2593 CE Den Haag
Postbus 93245
2509 AE Den Haag
T 070 349 5111
F 070 349 5100
info@zonmw.nl
www.zonmw.nl

contactpersoon
Geertje Appel
T 070 349 5259
F 070 349 5389
DoelmatigheidsOnderzoek@zonmw.nl

dossiernummer 945-17-102
uw kenmerk
ons kenmerk 2006/15693/ZONMW
datum 06 oktober 2006

onderwerp Honorering subsidieaanvraag, projectnummer 945-17-102

Geachte heer Widdershoven,

In onze brief van 29-06-2006 hebben wij u medegedeeld dat ZonMw uw subsidieaanvraag met de titel *Effect of small group quality improvement on prescribing and test ordering performance of general practitioners. A large scale implementation study in the South of the Netherlands (80-007028-98-07102)* zou honoreren nadat u ons de startdatum zou opsturen. Hieraan heeft u voldaan waardoor ik u met genoegen meedeel dat uw subsidieaanvraag is gehonoreerd. Bij verdere correspondentie en vragen aan ZonMw over het project vragen wij u uw projectnummer (945-17-102) te vermelden.

Financiering
De financiële bijdrage van ZonMw voor uw project bedraagt in totaal maximaal € 104.173 voor de duur van maximaal 36 maanden. Dit bedrag is inclusief eventueel verschuldigde BTW. De cofinanciering bedraagt € 195.083,-, waarbij een kopie van de brief waarin de cofinanciering is toegezegd inmiddels in ons bezit is. In onze brief van 29-06-2006 is een toelichting op de bedragen gegeven. De startdatum van uw project is 01-05-2006.

Aan de financiering zijn voorwaarden verbonden. Hiervoor verwijs ik u naar de bijgevoegde subsidievoorwaarden ZonMw, 1 juli 2004 en de aanvullende voorwaarden voor het programma DoelmatigheidsOnderzoek.

Het is belangrijk dat u ZonMw zo spoedig mogelijk, doch uiterlijk binnen 4 weken schriftelijk laat weten:

- Of u instemt met de voorwaarden die van toepassing zijn op de toekenning van de financiële bijdrage;
- De bank –en referentiegegevens die van toepassing zijn bij de betalingen van de subsidie.

Pas na uw instemming met de voorwaarden én na de daadwerkelijke start van uw project, zal ZonMw het voorschot voor het eerste projectjaar verstrekken. Over de hoogte van het bedrag en de betaaltermijnen zullen wij u nader informeren.

Heeft u naar aanleiding van deze brief vragen, neemt u dan gerust per e-mail of eventueel telefonisch contact op met de contactpersoon die in het briefhoofd vermeld staat. Ik verzoek u vriendelijk in uw communicatie met ZonMw steeds het projectnummer te vermelden.

Ik wens u veel succes met de uitvoering van uw project.

Met vriendelijke groet,
namens het bestuur,

Henk J. Smid
directeur

Kopie: projectleider en penvoerder
contactpersoon DoelmatigheidsOnderzoek
Jac's den Boer & Vink BV controller
F&C, ZonMw

Bijlagen: Subsidievoorwaarden ZonMw, 1 juli 2004
Aanvullende subsidievoorwaarden programma DoelmatigheidsOnderzoek

Wilt u uw mening geven, klagen of bezwaar maken?

1. U kunt (liefst schriftelijk) uw mening geven. ZonMw vat dit op als een signaal en behandelt uw opmerkingen zorgvuldig en serieus.
2. U kunt schriftelijk een klacht indienen. ZonMw heeft een speciale klachtenprocedure. De directie van ZonMw behandelt iedere schriftelijk ingediende klacht.
3. U kunt bezwaar maken tegen een beschikking. Stuurt u dan binnen zes weken na de dag waarop het besluit bekend is gemaakt een bezwaarschrift aan de Commissie beroep- en bezwaarschriften ZonMw, p/a Postbus 93 138, 2509 AC Den Haag.

ZonMw adviseert u de folder Signaleren, klagen en bezwaar maken te lezen (www.zonmw.nl). Deze kunt u gratis opvragen via ons algemeen telefoonnummer (070) 349 51 11 of per e-mail: info@zonmw.nl.

Het is raadzaam eerst contact op te nemen met de programmamedewerker van ZonMw (de contactpersoon). Niet altijd hoeft u voor het 'zware' middel van het bezwaarschrift te kiezen. In de praktijk blijken problemen en ontevredenheid rond de ZonMw-procedure vaak naar tevredenheid opgelost te worden na (telefonisch) contact met de programmamedewerker.

Aanvullende subsidievoorwaarden ZonMw DoelmatigheidsOnderzoek 2007

Naast de ZonMw subsidievoorwaarden juli 2004 zijn de volgende aanvullende voorwaarden van toepassing voor het programma DoelmatigheidsOnderzoek:

1. Aanvulling op artikel 6, lid 2
   In geval van outputfinanciering maakt de aangehechte bijlage met de door de subsidieontvanger geleverde opgave van de verwachte patiënteninstroom integraal onderdeel uit van de subsidievoorwaarden.

2. Aanvulling op artikel 6, lid 11 in geval van outputfinanciering
   Bij voortgangsverslagen van patiëntgebonden onderzoek gaat bijzondere aandacht uit naar de voortgang van de patiënteninclusie. Aan de hand van de door de subsidieontvanger geleverde opgave van de verwachte patiënteninstroom wordt de voortgang bewaakt. Indien de patiënteninclusie achterblijft bij de verwachtingen, dat wil zeggen minder dan 80% van hetgeen vooraf is afgesproken voor de betreffende periode, behoudt ZonMw zich het recht voor om het onderzoek te beëindigen. Bij tussentijdse beëindiging van het onderzoek kan ZonMw de verstrekte subsidievoorschotten terugvorderen.

3. Aanvulling op artikel 7, lid 3
   De zin 'In het eindverslag wordt de uitvoering van het VIP geëvalueerd.' vervalt. Eindverslag en definitieve VIP worden gelijktijdig ingestuurd. De subsidieontvanger ontvangt na inhoudelijke beoordeling van het eindverslag bericht over het VIP.

4. Bij gerandomiseerd patiëntgebonden onderzoek dient u uw studie aan te melden bij het Nederlands Trial Register (NTR). Zie voor meer informatie: www.trialregister.nl.
