# Supplementary material for: A cluster randomized controlled trial aimed at implementation of local quality improvement collaboratives to improve prescribing and test ordering performance of general practitioners: Study Protocol
Source: Implement Sci. 2009 Feb 17;4:6. doi: 10.1186/1748-5908-4-6 (PMC2656449; doi:10.1186/1748-5908-4-6)
Supplement: Additional file 5 — Approval ethical committee. Scanned letter of the Maastricht ethical committee, stating that it is not required to fully review this trial by the committee because Dutch law on research with humans is not applicable. [file 1748-5908-4-6-S5.pdf]

academisch ziekenhuis
Maastricht

P. Debyelaan 25
postbus 5800
6202 AZ Maastricht

telefoon 043-387 65 43
telefax 043-387 78 78

academisch ziekenhuis
Maastricht
azM

De heer J. Trietsch
Capaciteitsgroep Huisartsgeneeskunde
En
De heer M.P.W. Lamberti
Directeur CTCM

afdeling Medisch Ethische Commissie azM/UM
uw kenmerk
ons kenmerk MEC 06-4-033.2/pl
doorkiesnummer 043-387 6009
datum 24 augustus 2006
e-mail mecsecretariaat@ctcm.azm.nl

Betreft: **Effect of small group quality improvement on prescribing and test ordering performance of general practitioners. A large scale implementation study in the South of the Netherlands (MEC 06-4-033)**

Geachte heren,

De Medisch Ethische Commissie heeft bovengenoemd onderzoeksvoorstel besproken in haar vergadering van 23 augustus 2006.

Opgemerkt is dat het geen onderzoek in het kader van de WMO betreft. De Medisch Ethische Commissie heeft geen bezwaar tegen de uitvoering van bovengenoemd onderzoeksvoorstel en brengt derhalve een positief advies uit.

De commissie heeft de volgende stukken in haar toetsing betrokken:
- het protocol d.d. 13-02-2006;
- de toestemmingsverklaring d.d. 03-08-2006;
- de nadere toelichting d.d. 03-08-2006.

De commissie ontvangt nog graag bericht van de start- en einddatum van genoemde studie.

Aangezien voor de uitvoering van deze studie geen gebruik wordt gemaakt van faciliteiten van het azM, is het onderzoeker toegestaan te starten met deze studie.

Met vriendelijke groet,

namens de Medisch Ethische Commissie azM/UM,

mr. R.C.W. van Gils,
ambtelijk secretaris

dr. C.E.M. de Die-Smulders,
voorzitter
