# Supplementary material for: A cluster randomized controlled trial aimed at implementation of local quality improvement collaboratives to improve prescribing and test ordering performance of general practitioners: Study Protocol
Source: Implement Sci. 2009 Feb 17;4:6. doi: 10.1186/1748-5908-4-6 (PMC2656449; doi:10.1186/1748-5908-4-6)
Supplement: Additional file 6 — CONSORT Cluster RCT Checklist. checklist with reference to page numbers in this trial concerning the CONSORT statement for cluster-RCTs. [file 1748-5908-4-6-S6.doc]

**CONSORT Checklist of items to include when reporting a cluster randomised trial**

(Campbell MK, Elbourne DR, Altman DG: CONSORT statement: extension to cluster randomised trials. BMJ 2004, 328(7441):702-708.)

| **Paper section and topic** | **Item** | **Descriptor [section title]^1^** | **Page number** |
| --- | --- | --- | --- |
| **Title and abstract**  Design | 1 | How participants were allocated to interventions (eg random allocation, randomised, or randomly assigned), specifying that allocation was based on clusters **[Title; Abstract].** | 1-2 |
| **Introduction**  Background | 2 | Scientific background and explanation of rationale **[Background]**, including the rationale for using a cluster design **[Trial design]**. | 3 |
| **Methods**  Participants | 3 | Eligibility criteria for participants and clusters **[Inclusion criteria; Exclusion criteria]** and the settings and locations where the data were collected **[Recruitment of general practices; Recruitment of patient participants]**. | 7 |
| Interventions | 4 | Precise details of the interventions intended for each group, whether they pertain to the individual level, the cluster level, or both, and how **[Control group; Intervention group]** and when they were actually administered **[Timing of recruitment, intervention delivery and follow-up]**. | 9 |
| Objectives | 5 | Specific objectives and hypotheses and whether they pertain to the individual level, the cluster level, or both **[Trial objectives]**. | 5 |
| Outcomes | 6 | Report clearly defined primary and secondary outcome measures, whether they pertain to the individual level, the cluster level, or both **[Primary outcome measures; Secondary outcome measures; Table 1]**, and, when applicable, any methods used to enhance the quality of measurements (eg multiple observations, training of assessors) **[Data quality assurance]**. | 13 |
| Sample size | 7 | How total sample size was determined (including method of calculation, number of clusters, cluster size, a coefficient of intracluster correlation (ICC or k), and an indication of its uncertainty) and, when applicable, explanation of any interim analyses and stopping rules **[Sample size]**. | 9 |
| Randomization  Sequence generation | 8 | Method used to generate the random allocation sequence, including details of any restriction (eg blocking, stratification, matching) **[Randomisation and allocation concealment]**. | 8 |
| Allocation concealment | 9 | Method used to implement the random allocation sequence, specifying that allocation was based on clusters rather than individuals and clarifying whether the sequence was concealed until interventions were assigned **[Randomisation and allocation concealment]**. | 8 |
| Implementation | 10 | Who generated the allocation sequence **[Randomisation and allocation concealment**], who enrolled participants **[Recruitment of general practices; Recruitment of patient participants; Applying the eligibility criteria]**, and who assigned participants to their groups **[Randomisation and allocation concealment]**. | 8 |
| Blinding (masking) | 11 | Whether participants, those administering the interventions, and those assessing the outcomes were blinded to group assignment **[Blinding]**. If done, how the success of blinding was evaluated. | 7 |
| Statistical methods | 12 | Statistical methods used to compare groups for primary outcome(s) indicating how clustering was taken into account; methods for additional analyses, such as subgroup analyses and adjusted analyses **[Analyses]**. | 15 |

^1^ The bold test in square brackets refers to the section title within the publication.
